# Traitement chirurgical des lésions sacro-iliaques dans les fractures instables de l’anneau pelvien par vissage sacro-iliaque percutané

**DOI:** 10.11604/pamj.2016.24.168.8678

**Published:** 2016-06-28

**Authors:** Mohamed Amine Karabila, Ismail Hmouri, Tarik Madani, Younes Mhamdi, Mohamed Azouz, Mohamed Kharmaz, Mohamed Ouadaghiri, Moulay Omar Lamrani, Abdou Lahlou, Ahmed Bardouni, Mustapha Mahfoud, Mohamed Saleh Berrada, Christian Vasile

**Affiliations:** 1 Service de Chirurgie Orthopédique et de Traumatologie, CHU Ibn Sina, Rabat, Maroc; 2 Service de Chirurgie Orthopédique et de Traumatologie, Centre Hospitalier de Chambéry, France

**Keywords:** Instables, vissage, percutané, Unstable, screwing, percutaneous

## Abstract

Les fractures du bassin, le plus souvent multiples, sont fréquemment instables et surviennent le plus souvent dans des contextes traumatiques violents. Le traitement orthopédique de ces lésions est souvent pénible pour le patient et pour le personnel médical nécessitant une décharge au lit ou parfois des tractions qui peuvent aller jusqu'à 45 jours et peut compromettre la statique et la marche, le traitement chirurgical à ciel ouvert est un geste difficile, grevé d'une morbidité non négligeable avec un risque vasculaire (plexus veineux), nerveux (racines sacrées) ou septique notamment à prendre en compte, elle est donc généralement réservée aux formes neurologiques et fortement déplacées. La fixation percutanée sous radioscopie dans les fractures instables du bassin type B et C permet la synthèse des lésions postérieures source d’instabilité en fixant l’os coxal avec le corps de S1 ou S2 et la reprise rapide de la rééducation et de la marche.

## Abstract (en)

Pelvic fractures, usually are multiple, unstable fractures occuring most frequently due to violent traumatic events. Orthopedic treatment of these lesions is often distressing both to patients and medical personnel since it requires bed discharge or sometimes tractions which can have a duration up to 45 days and can compromise the static and the walking mechanisms; open operative management is difficult, carrying a significant burden in terms of morbidity and associated with vascular (venous plexus), nervous (sacred roots) or septic risk to take into account. For this reason it is generally reserved for patients with neurologic and strongly displaced forms. Percutaneous fixation under fluoroscopy in unstable type B and type C pelvic fractures allows the synthesis of posterior lesions causing instability by fixing the hip bone to S1 or S2 body, a rapid recovery and rehabilitation of walking.

## Introduction

Les fractures de l’anneau pelvien sont des urgences post-traumatiques vitales fréquentes généralement violentes, entrainant une instabilité de la ceinture pelvienne [1, 2]. Il s'agit le plus souvent d'accidents de circulation, de sport, plus rarement de chutes (montagne, travail, défenestration). Ces fractures représentent 5% des fractures et surviennent dans le cadre des polytraumatismes dans 20% et peuvent engager le pronostic vital du patient soit du fait de polytraumatisme soit du fait d’un saignement interne ou externe. Cette technique est peu traumatisante, elle améliore notablement la qualité de confort de l'opéré et la remise en fonction, réduisant à quelques jours l'hospitalisation postopératoire et elle génère en outre une économie considérable en terme de santé publique. Slatis et Huitiven [3] annoncent de mauvais résultats quand le défaut de réduction dépasse 2 cm. Rout considère comme acceptable un défaut de réduction de l'ordre de 1 cm. Donc si la réduction n’était pas obtenue en peropératoire, il faut convertir vers la chirurgie sanglante à foyer ouvert dès qu’il y ait un défaut de plus de 1 cm. Nous proposons de décrire la prise en charge radiologique interventionnelle et chirurgicale des ostéosynthèses des lésions sacro-iliaques instables par vissage percutané.

## Patient et observation

**Figure 1:**
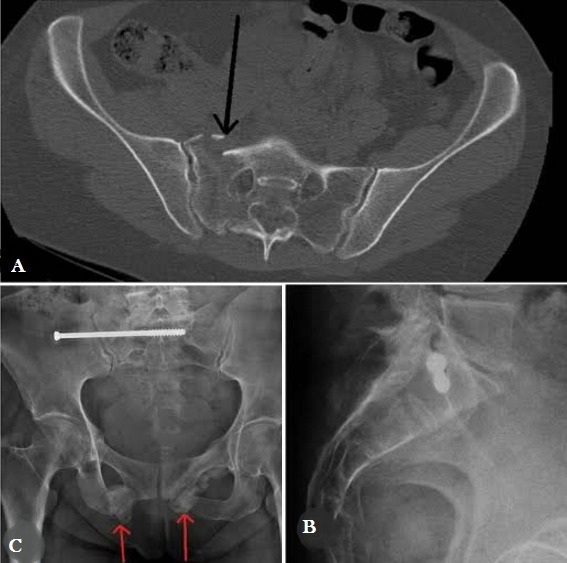
A) scanner du bassin montrant la fracture déplacée du sacrum; B) radiographie profil strict montrant le vissage sacro-iliaque; C) radiographie de face du bassin montrant la bonne consolidation du sacrum et des cadres obturateurs

**Cas 1:** Patiente âgée de 63 ans, victime d’une chute d’escalier occasionnant une fracture type C de Tile (fracture l’aileron sacré droit, des 2 cadres obturateurs) avec un trouble sensitif du membre inférieur droit type: engourdissement du gros orteil et des paresthésie au niveau du triceps sural, à j+2 elle a bénéficié d’un vissage sacro-iliaque droit par une vis perforée diamètre 7mm et de 110 de longueur avec filtrage de 32 mm. A 15 mois de recul, la fracture est consolidée (Figure 1) et la patiente a repris ses activités sans limite et sans douleur (régression totale des troubles sensitifs).

**Figure 2:**
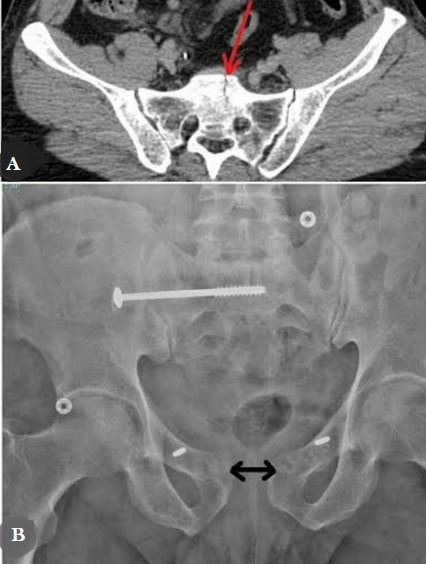
A) scanner du bassin montrant la fracture du sacrum; B) radiographie de face du bassin après 6 mois montrant la bonne consolidation du sacrum et le petit distasis au niveau de la symphyse pubienne

**Cas 2:** Patient âgée de 55 ans, qui présente suite à une chute de parapente une fracture type B1 de Tile (fracture du sacrum peu déplacée et disjonction pubienne estimée à 3 cm). Après stabilisation se son état hémodynamique, le patient était opéré à j+3 par une plaque antérieure et un vissage sacro-iliaque avec bonne évolution clinique. 4 mois après, le patient a présenté une infection en regard de la symphyse ce qui a nécessité une ablation de la plaque avec persistance d’un petit diastasis au niveau de la symphyse pubienne (Figure 2) sans répercussion fonctionnelle majeure sur la statique et la marche du patient.

**Figure 3:**
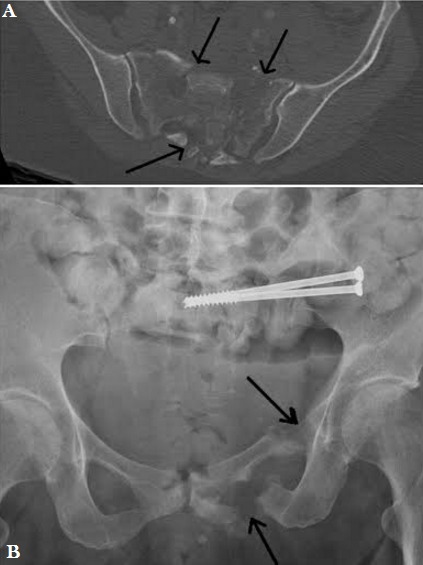
A) fracture complexe du sacrum; B) contrôle après 8 mois montrant la consolidation du sacrum et la pseudarthrose de la branche ischio-pubienne

**Cas 3:** Patiente de 62 ans, polytraumatisée victime d’une chute de 10 mètres suite à une tentative de suicide, vue aux urgences pour fracture du poignet gauche, fracture du calcanéum gauche et fracture du bassin type C de Tile (cadre obturateur gauche, fracture complexe de l’aileron sacré gauche) avec des signes neurologiques au niveau du membre inférieur type paresthésie et elle a eu comme ostéosynthèse: embrochage du poignet, plaque du calcanéum et vissage par 2 vis canulées de diamètre 7.5 mm, filetage 32 mm et de taille 105 et 110 mm puis elle a été mise en décharge pendant un mois. Après 8 mois d’évolution, la patiente elle marchait sans béquilles mais avec une légère boiterie et à la radiographie on constatait une pseudarthrose de la branche ischio-pubienne (Figure 3).

**Figure 4:**
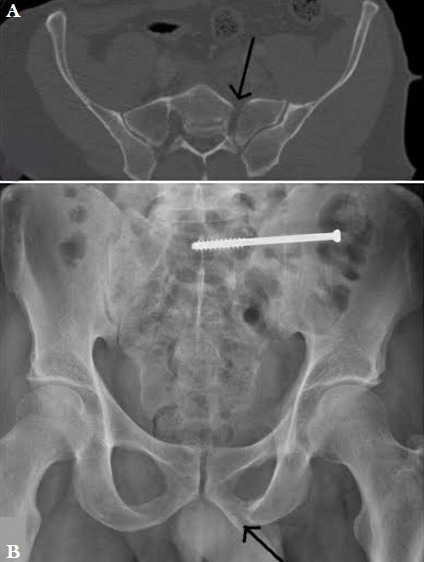
A) fracture verticale déplacée du sacrum; B) contrôle radiologique après 10 mois montrant la consolidation du sacrum et du cadre obturateur

**Cas 4:** Il s'agit d'un patient de 41 ans, qui a sauté d'un deuxième étage en état d'ébriété, pour s'échapper d'une pièce où il était enfermé. il présente une fracture diaphysaire de l’humérus gauche, luxation du coude, fracture comminutive de radius distal et une fracture du bassin type C de Tile (aile iliaque gauche, aileron sacré gauche, branches ilio et ischio pubiennes gauches). Il a bénéficié d’une ostéosynthèse de l’humérus et du poignet et d’un vissage sacro-iliaque par une vis perforée 90 mm, de diamètre 7 et 32 mm de filetage. A 10 mois de recul, le foyer est consolidé (Figure 4) et le patient a repris ses activités et peut marcher 10 kilomètres.

**Figure 5:**
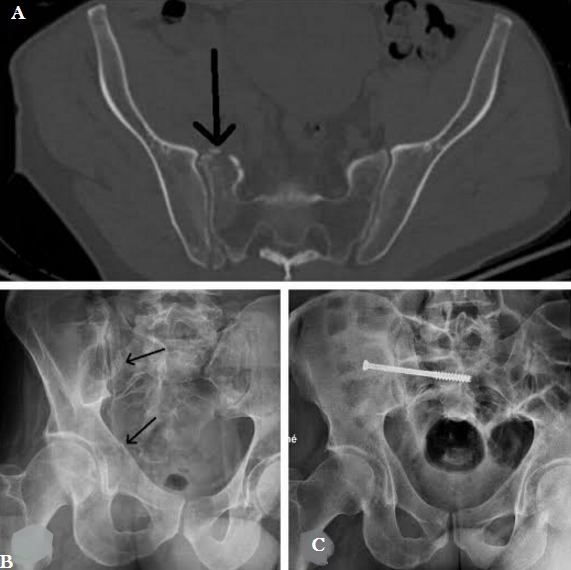
Fracture Tile B légèrement instable traitée par vissage

**Cas 5:** Patiente âgée de 51 ans, victime d’une chute mécanique en compression latérale et présente sur le plan radiologique une fracture type B instable de Tile (fracture de l’aileron sacré et du cadre obturateur droit) ostéosynthésée par vissage percutané avec une très bonne évolution clinique et radiologique sans déplacement secondaire (Figure 5). A 12 mois de recul, elle peut marcher jusqu’à 15 kilomètres en pente avec du poids au dos.

## Discussion

Le traitement chirurgical classique des fractures instables du bassin est très invasif et pose des difficultés de réduction et de contention. La problématique de la chirurgie à ciel ouvert est de fixer l’os iliaque au sacrum en évitant le délabrement musculaire et les pertes sanguines. L’avènement de la radiologie interventionnelle et en particulier du scanner interventionnel a permis par des micro-incisions de positionner des vis à travers l’os iliaque et le sacrum par un guidage très précis assurant une stabilisation satisfaisante sans délabrement musculaire. La fixation externe en urgence par le fixateur de Hoffman, la pose d'un pantalon antichoc ou un clamp pelvien et plus simplement une traction trans-osseuse permettent dans ces cas de comprimer le bassin et ou de stabiliser les caillots permettant ainsi de franchir le cap de l'urgence initiale, puis de procéder aux explorations nécessaires. L'intérêt de cette technique percutanée a été bien décrit dans la littérature avec en particulier une nette diminution des pertes sanguines et du risque d'infection [4, 5]. L'appréciation des lésions postérieures dans les lésions post-traumatiques de la ceinture pelvienne est souvent sous-évaluée, en raison de la complexité anatomique de la région, mais surtout des superpositions digestives majorées par l'iléus réactionnel fréquemment présent. C'est pourquoi, la tomodensitométrie (TDM) doit être rapidement mise en œuvre, étant plus précise dans l'analyse des lésions de l'arc postérieur [6]. Parmi les classifications des fractures du bassin les plus communément admises, nous retenons celle de Tile et Pennal [7], fondée sur la stabilité dans le plan vertical et sur la direction des forces (Tableau 1): compression antéro-postérieure (APC); compression latérale (LC); mécanisme de cisaillement vertical.

**Tableau 1:** Classification de Tile pour les fractures de l’anneau pelvien

Type A: stable	Type B: partiellement stable	Type C: instable
**A1** Fractures n’interrompant pas la continuité de l’anneau pelvien (aile-tubérosités)	**B1** Disjonction pubienne (open book)	**C1** Lésion unilatérales
**A2** Fractures stables peu déplacées de l’anneau pelvien	**B2** Compression latérale homolatérale	**C2** Lésion bilatérales
	**B3** Compression bilatérale	**C3** Lésions associées à une fracture du cotyle

La procédure est réalisée sous anesthésie générale en position de décubitus dorsal avec un arceau de scopie manipulé par le chirurgien et le technicien radiologue. Le matériel utilisé comporte une broche guide, un marteau chirurgical, une mèche pour trajet de vis et des vis canulées taille 7,5mm et un moteur. La réduction se fait en appliquant une traction dans l’axe du membre puis le repérage du point d’entrée se fait sur un cliché de profil strict du sacrum et la progression de l’ostéosynthèse est contrôlée régulièrement par les incidences d’inlet et d’outlet. La réduction des lésions avant toute tentative de vissage est indispensable. Si la réduction n'est pas obtenue, il est préférable de s'orienter vers une autre technique d'ostéosynthèse. Cette exigence pour Rommens devrait inciter à ce que seuls les chirurgiens capables de faire une ostéosynthèse à foyer ouvert entreprennent l'opération percutané [8]. La chirurgie doit se faire le plus rapidement possible après le traumatisme et si possible dans les 24 premières heures. Elle peut même s'effectuer en urgence s'il existe une défaillance circulatoire et remplace avantageusement la mise en place d'un fixateur externe. Les fractures de type B ne posent en principe pas de véritable problème de prise en charge actuellement. Une simple traction dans l’axe de fémur est généralement suffisante pour obtenir la réduction du déplacement horizontal du sacrum ou de la sacro-iliaque. Dans les fractures instables type C qui s’accompagnent d’une instabilité complète postérieure et antérieure, la réduction des lésions postérieures entraîne très souvent une réduction des lésions antérieures, pourtant un complément d'ostéosynthèse antérieure est nécessaire s’il y a un diastasis dépassant 2,5 cm au niveau de la symphyse. L'ostéosynthèse des fractures du cadre obturateur n'est pas utile et une simple décharge au lit aboutira à la consolidation. Une stabilité sacro-iliaque optimale est obtenue lorsque l’extrémité de la vis dépasse la ligne médiane du sacrum et se termine au niveau de sa corticale antérieur. La rééducation, quand elle est possible, peut être immédiatement initiée. Le patient pourra recommencer la marche au bout d’une semaine avec une ceinture pelvienne ce qui permet d’éviter les complications de décubitus prolongé. Le vissage percutané n'est pas un but en soi. Il nous semble plus important d'insister sur la qualité de la réduction obtenue, le vissage percutané n'étant alors utile que pour maintenir cette réduction. Si la réduction n'est pas obtenue par manœuvre externe, il faudra privilégier une autre technique de réduction.

## Conclusion

Le vissage sacro-iliaque percutané nous semble une technique fiable et reproductible. Le traitement des fractures instables du bassin (type C de l'AO) est pour nous la meilleure indication. Maintenant beaucoup de publications sur le sujet s'intéressent actuellement sur les moyens électromyographies ou tomodensitométriques préopératoires permettant un vissage plus sûr en limitant les risques vasculo-nerveux [9, 10].
